# Integrated Structured Breakfast and Morning Sport Program and Its Associations with Attention, Executive Functions, and Academic Performance in Students

**DOI:** 10.3390/nu18132103

**Published:** 2026-06-27

**Authors:** Francesca Latino, Domenico Tafuri, Emma Saraiello, Maria Giovanna Tafuri

**Affiliations:** 1 Department of Biological and Environmental Sciences and Technologies, University of Salento, 73100 Lecce, Italy; francesca.latino@unisalento.it; 2 Department of Medical, Human Movement and Well-Being Sciences, University of Naples “Parthenope”, 80100 Naples, Italy; domenico.tafuri@uniparthenope.it; 3 Department of Literary, Linguistic and Philosophical Studies, Pegaso University, 80143 Naples, Italy; mariagiovanna.tafuri@unipegaso.it

**Keywords:** adolescence, breakfast, cardiorespiratory fitness, cognitive activation, executive functions, school-based intervention, sport

## Abstract

Background/Objectives. Increasing evidence suggests that nutrition and sport participation may positively influence cognitive functioning, readiness for learning, and academic achievement during adolescence. However, limited research has investigated the combined effects of structured breakfast programs and cognitively oriented sport-based interventions implemented in real school settings. The present study aimed to examine the associations between participation in an integrated school-based program consisting of structured breakfast and morning sport sessions and executive functions, physiological well-being, school engagement, and academic achievement in adolescent students. Methods. A 16-week quasi-experimental pre–post study with class-based allocation was conducted in a secondary school in Southern Italy. A total of 110 students aged 14–16 years participated in the study. The experimental group, comprising 55 students, participated in a structured breakfast program combined with cognitively oriented morning sport-based sessions conducted three times per week for 40 min before regular lessons, whereas the control group continued ordinary school activities. Cognitive assessment included the Stroop Color and Word Test and the Digit Span Test in both forward and backward conditions. Physiological measures included body mass index, resting heart rate, and the 20 m shuttle run test. Nutritional habits, school engagement, and academic achievement were also evaluated through questionnaires and school records. Results. Compared with the control group, students participating in the integrated program showed more favorable changes in selective attention, inhibitory control, working memory performance, cardiorespiratory fitness, breakfast habits, and school engagement over the study period. Moderate positive changes in academic achievement were also observed, whereas no substantial anthropometric changes emerged during the study period. Conclusions. The findings suggest that participation in an integrated school-based program combining structured nutrition and cognitively oriented sport activities was associated with improvements in cognitive functioning, healthy habits, and academic outcomes during adolescence. These findings highlight the potential value of multidimensional educational approaches integrating health promotion and learning processes within school environments.

## 1. Introduction

In recent years, international literature has devoted increasing attention to the relationship between health, learning, and cognitive functioning within school settings [1]. In particular, numerous studies have highlighted how dietary habits and participation in structured sport activities may significantly influence not only students’ general health status, but also the cognitive processes involved in learning, behavioral regulation, and academic performance [2]. Within this perspective, schools are increasingly considered not merely as environments devoted to the transmission of disciplinary knowledge, but also as privileged contexts for promoting healthy lifestyles and fostering cognitive, emotional, and relational competencies that are functional to educational success. This perspective is particularly relevant during adolescence, a developmental stage characterized by profound biological, psychological, and social transformations that influence both eating behaviors and attentional, motivational, and executive capacities [3].

Among the factors most strongly associated with adolescents’ cognitive and academic performance, breakfast plays a central role. Scientific literature has extensively documented how the regular consumption of a balanced breakfast is associated with improved attention, working memory processes, information processing speed, and concentration during school hours. Regular breakfast consumption has been associated with improved attention, working memory, concentration, and academic performance, whereas breakfast skipping has frequently been linked to reduced cognitive efficiency, lower school engagement, and less favorable educational outcomes [4]. These findings become even more relevant considering that adolescence represents a developmental phase in which eating habits often become irregular, influenced by accelerated lifestyles, limited time devoted to meals, and increasing consumption of calorie-dense but nutritionally poor foods [5]. At the same time, participation in structured sport activities has progressively been recognized as a potentially important factor in supporting cognitive functioning and academic achievement. Unlike simple spontaneous or recreational physical activity, sport-based activities involve highly complex motor, cognitive, decisional, and relational components [6]. Sport requires continuous action planning, inhibitory control, rapid adaptation to environmental stimuli, attentional management, and real-time decision making. These characteristics make sport experience particularly relevant within studies focusing on executive functions [7].

Executive functions encompass a set of higher-order cognitive processes that enable individuals to regulate goal-directed behavior. These include inhibitory control, working memory, cognitive flexibility, planning, and the ability to maintain attention during complex tasks [8]. Numerous studies have shown that regular involvement in structured sport activities may contribute to the enhancement of these functions, particularly during developmental age. In particular, sports characterized by high coordinative demands, rapid decision making, and dynamic interaction with the environment have been associated with improvements in cognitive processing speed, selective attention, self-regulation capacities, and executive components of working memory [9]. These benefits have been attributed to physiological and neurocognitive adaptations associated with regular sport participation [10]. Furthermore, participation in sport activities within school contexts may positively influence motivational and relational dimensions of learning, increasing student engagement, self-efficacy, and active participation in school life [11].

Within this framework, the concept of readiness for learning becomes particularly relevant, understood not merely as academic preparation, but as a global condition of cognitive, emotional, and physiological readiness for learning [12]. The ability to effectively cope with school demands depends on multiple interconnected factors, including sleep quality, nutrition, emotional regulation, energy levels, and cognitive activation status. Early morning hours represent a particularly delicate phase, as students must progressively reach optimal levels of attention, concentration, and cognitive readiness in order to successfully engage in academic activities. In this regard, the combination of a balanced breakfast and a morning sport session has been proposed as a potentially valuable educational strategy for supporting neurocognitive activation before the beginning of classes. Morning sport practice, especially when characterized by coordinative exercises, integrated cognitive activities, and dynamic motor situations, may contribute to increasing alertness, improving mood, and facilitating attentional processes [13,14]. At the same time, the nutritional support provided by breakfast may ensure an adequate energy supply to the brain, sustaining cognitive stability during subsequent school activities.

Despite growing interest in the relationship between nutrition, sport participation, and learning, important gaps remain. Most studies have examined breakfast habits and sport activity separately [15,16,17,18,19,20,21,22,23], whereas relatively few investigations have explored their combined effects within integrated school-based interventions. Furthermore, existing research has frequently focused on general physical activity rather than cognitively demanding sport-based activities [24,25,26,27], and only a limited number of studies have simultaneously considered nutritional, cognitive, physiological, and academic outcomes within real school settings [28,29]. Evidence is particularly scarce regarding programs that combine a structured breakfast with cognitively oriented morning sport sessions implemented immediately before the beginning of regular lessons [30]. Consequently, further research is needed to examine whether integrated and ecologically sustainable interventions of this type may contribute to improved readiness for learning, executive functioning, and academic performance among adolescents.

In light of these considerations, the present study aims to examine the associations between participation in an integrated school-based program consisting of a structured breakfast and morning sport sessions and executive functions, readiness for learning, and academic achievement among adolescent students. The intervention was designed according to a multidimensional perspective, simultaneously considering nutritional, cognitive, physiological, and educational aspects. The main hypothesis of the study is that students participating in the integrated program would demonstrate greater improvements in attention, executive functions, working memory performance, and readiness for learning over time compared to students following their usual school routine. Furthermore, it is hypothesized that favorable changes in these domains would be accompanied by improvements in academic performance indicators and students’ perceived well-being. More specifically, it is assumed that the combination of nutritional support and sport participation may be associated with more favorable learning conditions and may provide useful indications for the development of educational models oriented toward health, cognitive functioning, and academic success.

## 2. Materials and Methods

### 2.1. Study Design

The present study was designed as a quasi-experimental school-based intervention with pre- and post-intervention assessment, aimed at examining the associations between participation in a structured breakfast and morning sport program and executive functions, readiness for learning, and academic achievement among adolescent students. The study lasted 16 weeks and was conducted in a selected secondary school located in Southern Italy, chosen according to the institution’s willingness to participate in the project and the availability of adequate spaces for the implementation of the intervention protocol. The intervention program was carried out three times per week during the morning hours before the beginning of regular school lessons. On intervention days, students first consumed the structured breakfast, followed by a brief transition period and a 40 min cognitively oriented sport session. The experimental group (EG) participated in an integrated program including a structured breakfast and cognitively oriented sport-based activities aimed at promoting cognitive and neurophysiological activation, whereas the control group (CG) continued its ordinary school routine without intentional modifications to its morning habits.

Assessments were conducted at two different time points: a baseline phase before the beginning of the intervention and a post-intervention phase at the end of the 16-week program, using standardized procedures for both groups. The study was developed within a real school setting in order to ensure high ecological validity and to verify the organizational feasibility of a model potentially applicable to everyday educational practice. All procedures were conducted in accordance with the ethical principles of the Declaration of Helsinki for research involving human participants. The study protocol was approved by the Ethics Committee of the University of Naples Parthenope, Department of Medical, Motor and Wellness Sciences (DiSMMeB Prot. No. 88592/2024).

### 2.2. Participants

The study sample consisted of a total of 110 students attending the second year of secondary school, aged between 14 and 16 years (M = 15.1; SD = 0.7). The experimental group consisted of two classes including a total of 55 students, whereas the control group included two additional second-year classes with a total of 55 students. Group allocation was performed while fully preserving the natural composition of the pre-existing school classes, avoiding individual redistribution of students. This methodological choice was adopted in order to preserve the relational, organizational, and educational balance of the class groups, reduce possible interference with ordinary school activities, and ensure greater ecological validity of the intervention. Furthermore, maintaining the original class composition allowed the researchers to limit possible contamination effects deriving from artificial modifications of group structures or alterations in the social dynamics naturally characterizing the school environment. The four participating classes were selected from the same school grade within the same secondary school and followed the same educational curriculum. Class selection was based on organizational feasibility and compatibility with the intervention schedule established in collaboration with the school administration. Consequently, all classes shared the same school context, curricular requirements, and timetable structure. Baseline analyses further revealed no statistically significant differences between groups in the principal anthropometric, cognitive, physiological, nutritional, and academic variables assessed before the intervention. As allocation was based on pre-existing school classes rather than individual randomization, the study design preserved the natural educational setting while maintaining comparability between groups on the measured baseline characteristics.

Students were included in the study if they regularly attended the participating classes, their parents or legal guardians provided signed informed consent, and they provided assent to participate in the study. Additional inclusion criteria included eligibility for school sport participation according to current regulations, regular school attendance, and the ability to participate in all activities included in the protocol without significant physical or cognitive limitations. Exclusion criteria included medical contraindications to sport participation, metabolic or nutritional disorders requiring specific dietary regimens incompatible with the proposed breakfast program, severe neurological, cognitive, or psychiatric disorders potentially interfering with the execution of the planned assessments, prolonged absences during the intervention period, or failure to obtain informed consent. Before the beginning of the study, descriptive information regarding age, sex, body mass index (BMI) percentile classification, breakfast habits, and general health conditions declared by participants was collected. Information regarding extracurricular sport participation was explored only for preliminary descriptive purposes and was not included among the variables considered in the statistical analyses. A priori power analysis was performed using G*Power version 3.1.9.7 [31] software for repeated-measures ANOVA (within–between interaction). The analysis was based on the primary cognitive outcomes of the study, particularly executive function measures assessed through the Stroop Test and Digit Span tasks. Assuming a moderate effect size (f = 0.25), an alpha level of 0.05, statistical power of 0.80, two groups, and two repeated measurements, the minimum required sample size was estimated to be approximately 98–106 participants. Therefore, the final sample of 110 students, with 55 in the experimental group and 55 in the control group, was considered adequate to detect moderate Group × Time differences over the study period. Four additional participants were included beyond the minimum estimated sample size in order to reduce the risk of study attrition and potential participant dropout during the intervention period.

### 2.3. Procedures

The study procedures were organized in collaboration with school staff, physical education teachers, and professionals with nutritional expertise involved in the project. During the preliminary phase, students received detailed information regarding the objectives of the study, participation procedures, and assessment protocols through explanations adapted to their age and in accordance with the ethical principles of educational research. Pre-intervention assessments were conducted during school hours, preferably during the early morning and before the beginning of regular academic lessons, in order to standardize assessment conditions. At the end of the 16-week intervention period, the same assessment procedures were repeated following the same administration order to reduce possible procedural bias. Baseline assessments were conducted on the day preceding the start of the intervention period, whereas post-intervention assessments were performed on the day following the final intervention session. Consequently, the study was designed to evaluate changes associated with the entire 16-week intervention period rather than the acute effects of a specific breakfast or sport session. Assessors responsible for cognitive, physiological, and fitness evaluations were blinded to participants’ group assignment throughout both assessment phases. Statistical analyses were performed using anonymized coded datasets in order to maintain blinding during data processing and analysis.

Throughout the entire study period, adherence to the program was monitored through attendance registers for sport sessions, observation sheets, and brief diaries concerning the consumption of the structured breakfast, completed by students under teacher supervision. Sport-based activities were conducted within the school environment by qualified physical education teachers and supervised by the research team, whereas nutritional monitoring was performed through periodic assessments regarding breakfast habits and the quality of foods consumed by participants in the experimental group.

### 2.4. Measures

The evaluation of the intervention effects was carried out through a multidimensional approach including cognitive, physiological, nutritional, and academic measures, to comprehensively analyze the possible changes associated with the sport and nutrition program. All assessments were conducted both during the pre-intervention phase and at the end of the 16-week program, following standardized administration procedures and maintaining the same order of assessments between baseline and post-test evaluations.

Regarding cognitive assessment, the main components of executive functions directly related to readiness for learning and academic achievement were analyzed. Selective attention, inhibitory control, and information processing speed were assessed through the Stroop Color and Word Test [32], widely used in adolescent populations for the evaluation of attentional and executive processes. The test consists of three different conditions, word reading, color naming, and color–word interference, in which participants are required to identify the color of the ink while ignoring incongruent verbal information. Assessment was based on a composite performance score integrating completion time and number of errors across the test conditions. This composite score was used to simultaneously consider processing speed and task accuracy. Lower scores indicated better performance, reflecting shorter completion times and fewer errors. All Stroop Test scores were analyzed as raw composite values; no standardization procedures or z-score transformations were applied. Working memory was evaluated through the Digit Span Test [33] in both forward and backward conditions. In the forward condition, students were required to repeat numerical sequences in the same order of presentation, whereas in the backward condition they were required to repeat the sequences in reverse order. Assessment was based on the maximum number of correctly repeated sequences, with higher scores associated with better short-term information maintenance and manipulation abilities. Alternative stimulus sequences were used between baseline and post-intervention assessments to minimize potential practice effects associated with repeated testing. In addition, test administrators were blinded to participants’ group assignment throughout both assessment phases in order to reduce potential assessment bias.

Physiological assessment included anthropometric parameters and functional indicators associated with general well-being and neurophysiological activation. Body weight and height (Seca 877 digital scale and Seca 217 portable stadiometer, Seca GmbH & Co. KG, Hamburg, Germany) were measured using calibrated instruments for the calculation of body mass index expressed in kg/m^2^, with interpretation additionally based on age- and sex-adjusted BMI percentile classifications according to World Health Organization reference standards for adolescents [34]. Resting heart rate was recorded after at least five minutes in a seated position under controlled environmental conditions using digital heart rate monitors. Cardiorespiratory fitness was assessed through the 20 m shuttle run test [35], selected for its scientific validity and its widespread use in school and sport settings. The test consists of repeated back and forth running over a distance of 20 m following progressively faster audio signals. The test ends when the participant is no longer able to maintain the required pace, allowing an indirect estimation of aerobic capacity through the total number of completed levels.

Nutritional aspects were monitored through a brief questionnaire regarding morning eating habits, adapted from previously used dietary habits questionnaires developed for adolescent populations and inspired by the HELENA study framework [36], to analyze breakfast frequency, general quality of consumed foods, and hydration level during the early hours of the day. The questionnaire included multiple-choice questions concerning weekly breakfast frequency, types of foods usually consumed, intake of sugar-sweetened beverages, presence of fruit or whole-grain cereals, and water consumption during the morning. Each item was scored on a five-point scale, with higher values reflecting healthier breakfast-related behaviors. Item scores were equally weighted and averaged to obtain an overall Breakfast Habits Score ranging from 1 to 5, with higher scores indicating healthier and more regular breakfast behaviors. For the experimental group, adherence to the proposed nutritional program was additionally monitored through a brief weekly food diary completed by students under teacher supervision. The diary included a daily record of foods consumed during breakfast, beverage intake, and meal timing. An example of a diary entry included items such as “milk”, “whole grain bread”, “fresh fruit”, and “water”, together with the indication of the day of the week and breakfast time. The questionnaire was administered in Italian. Because the instrument was developed as a brief questionnaire based on previously published dietary habit frameworks rather than as a direct translation of a specific validated scale, no formal translation or cross-cultural adaptation procedure was required.

Academic outcomes included indicators related to academic achievement and school engagement. Academic achievement was assessed through students’ overall grade average across all curricular subjects included in the school curriculum, collected before and after the intervention. Academic grades were expressed according to the Italian grading system, which ranges from 1 to 10, with 6 representing the minimum passing grade and 10 indicating excellent performance. The participating classes belonged to the same educational track and were taught by the same teaching staff according to the same curricular requirements and school assessment procedures. Teachers were not informed about group allocation during the study period. Furthermore, a brief school engagement scale composed of eight items evaluated through a five-point Likert scale, ranging from 1 (“never”) to 5 (“always”), was administered. The scale was adapted from previous school engagement frameworks developed for adolescent populations, particularly the multidimensional model proposed by Fredricks et al. [37], and was designed to assess school motivation, attention during lessons, participation in classroom activities, and perceived involvement in everyday educational activities. The eight items explored students’ ability to maintain attention during lessons, active participation in classroom activities, involvement in school tasks, interest in learning activities, motivation toward school attendance, participation in classroom discussions, commitment to academic tasks, and perceived engagement in everyday school life. Examples of items included statements such as “I am able to maintain attention during lessons”, “I actively participate in school activities”, and “I feel involved in classroom activities”. The final score was calculated as the mean of the eight items, resulting in values ranging from 1 to 5, with higher scores indicating greater perceived school engagement. Internal consistency of the scale was evaluated using Cronbach’s alpha coefficient. The school engagement scale was administered in Italian. The items were reviewed by the research team to ensure linguistic clarity and contextual appropriateness for the participating students. As the instrument did not represent a direct translation of a specific validated scale, no formal translation or cross-cultural adaptation procedure was performed.

### 2.5. Intervention

The intervention (Table 1) was developed according to an integrated approach aimed at combining nutritional education, neurophysiological activation, and morning sport practice within the school setting. The program lasted 16 consecutive weeks and involved only the experimental group. Activities were conducted three times per week during the hours preceding regular academic lessons, through sessions lasting a total of 40 min. On intervention days, students first consumed the structured breakfast (typically requiring approximately 10–15 min), followed by a brief transition period. Subsequently, they participated in a 40 min cognitively oriented sport session. Regular academic lessons began immediately after completion of the intervention activities. The sequencing of breakfast, exercise, and academic lessons was consistent with previous evidence indicating that the combination of breakfast consumption and morning exercise may support cognitive readiness and academic functioning during the early hours of the school day, as reported by Kawabata et al. [38]. Regular academic lessons began immediately after completion of the intervention activities. Each session included two integrated components. The first component consisted of a structured breakfast organized within the school environment and supervised by the personnel involved in the project. The second component included cognitively oriented sport-based activities designed to stimulate cognitive, attentional, and neurophysiological activation before the beginning of school lessons.

The structured breakfast was designed according to nutritional principles consistent with dietary guidelines for adolescence and with the Mediterranean dietary model (Table 2). The breakfast protocol was developed in collaboration with a qualified nutritionist and was specifically designed to provide adequate energy availability before the subsequent sport session and academic activities. Portion sizes were standardized across participants according to age-related nutritional recommendations and were planned to ensure a balanced intake of carbohydrates, proteins, fats, fiber, and hydration. The nutritional composition of the breakfast was monitored throughout the intervention period, and adherence was supervised by the nutritionist and project staff. Meals were characterized by a balance of complex carbohydrates, light proteins, fiber, and adequate hydration, avoiding foods with high levels of simple sugars or ultra-processed products. The nutritional plan included a weekly rotation of different meal combinations to ensure dietary variety and adequate energy intake during the early hours of the day. On the first weekly session, breakfast included partially skimmed milk or plain yogurt, whole-grain bread with honey or sugar-free jam, one portion of fresh fruit, and water. On the second weekly session, breakfast consisted of Greek yogurt or milk, whole-grain cereals, small portions of dried fruit, a banana or apple, and water. On the third weekly session, the meal included toasted whole-grain bread, ricotta cheese or low-fat fresh cheese, seasonal fresh fruit, and water. Portion sizes were adapted according to participants’ age and supervised by the staff involved in the project. The objective of the structured breakfast was to provide adequate energy and metabolic support during the early hours of the school day, with the intention of supporting glycemic stability, reducing morning cognitive fatigue, and facilitating attentional readiness.

The sport sessions were designed by prioritizing activities characterized by high coordinative demands, rapid decision making, selective attention, working memory engagement, and motor adaptation, while avoiding excessively intense exercises or activities exclusively focused on physical performance. Previous studies have suggested that modified and game-based motor activities may facilitate cognitive and motor engagement across developmental age groups [39]. Accordingly, the structure of the intervention assumed that cognitively demanding sport activities may be associated with neurophysiological activation processes relevant to school learning. Each session was organized into three main phases. The initial phase, lasting approximately 10 min, included general activation and dynamic coordination activities consisting of joint mobility exercises, light running, coordinative drills, dynamic balance exercises, and simple motor tasks associated with cognitive stimuli. The central phase, lasting approximately 20 min, included sport-based activities differentiated according to the objectives of the specific training day. The final phase, lasting approximately 10 min, was dedicated to active cool-down, controlled breathing, dynamic stretching, and brief attentional regulation activities.

The activities included in the central phase were organized according to a stable weekly schedule maintained throughout the entire intervention period in order to ensure methodological continuity and gradual progression of cognitive and coordinative demands.

During the first weekly session, activities were mainly oriented toward the development of motor coordination and selective attention. Students performed coordinative pathways involving changes in direction, speed exercises based on visual and auditory signals, ladder drills, motor reaction activities, and circuits with multiple attentional demands. Some exercises included the association between movement and simple cognitive tasks, such as recognizing colors, numbers, or auditory stimuli during motor execution.

During the second weekly session, the intervention focused on collaborative sport activities and motor decision making. Students participated in small-sided games, pair exercises, and cooperative motor activities aimed at stimulating rapid decision making, motor problem solving, adaptation to environmental stimuli, and attentional control in dynamic situations. Simplified sport games with variable rules and rapid adaptation requests introduced by the teacher were used.

During the third weekly session, activities were mainly oriented toward the integration of moderate aerobic endurance and cognitive tasks. Students performed intermittent moderate-intensity circuits, station-based activities, and continuous movement exercises integrated with memory tasks, motor sequences, and rapid responses to verbal or visual stimuli. The objective of this session was to simultaneously promote moderate cardiovascular activation and executive function engagement.

Throughout the intervention, a gradual progression of coordinative and cognitive demands was implemented by progressively increasing motor complexity, execution speed, and attentional processing requirements. Exercise intensity was monitored throughout the intervention using the OMNI Rating of Perceived Exertion (OMNI-RPE) [40] scale, a validated instrument for children and adolescents. Participants periodically reported their perceived exertion during the sport sessions, and the mean OMNI-RPE score recorded across the intervention was 5, indicating a moderate level of exercise intensity. The overall intensity of the activities was maintained at a moderate level, as confirmed by OMNI-RPE monitoring, in order to avoid fatigue conditions incompatible with subsequent participation in regular academic lessons and to prioritize cognitive activation rather than athletic overload.

All sessions were conducted by qualified teachers in the field of sport sciences and supervised by the research team. During the intervention period, students’ participation was monitored through attendance registers and observation sheets regarding engagement, adherence to activities, and compliance with nutritional indications.

The control group continued the ordinary school activities included in the educational curriculum and did not participate in the structured nutritional program provided to the experimental group. During the 16-week intervention period, both groups participated in three school-based physical activity sessions per week. The experimental group attended the structured cognitively oriented sport sessions integrated with the breakfast intervention, whereas the control group participated in regular physical education activities of comparable weekly frequency. Students in the control group did not receive specific nutritional indications, and no intentional modifications to their daily school routine were introduced during the study period.

### 2.6. Statistical Analysis

Statistical analyses were conducted using the Statistical Package for the Social Sciences, SPSS version 30.0 (IBM Corp., Armonk, NY, USA), with the level of significance set at *p* < 0.05. In a preliminary phase, all variables were subjected to descriptive analysis through the calculation of means, standard deviations, frequencies, and percentages, in order to describe sample characteristics and verify data distribution. Normality of distributions was assessed using the Shapiro–Wilk test, whereas homogeneity of variances was verified through Levene’s test.

To analyze baseline differences between the experimental and control groups regarding anthropometric, cognitive, physiological, and academic variables, independent-samples *t*-tests were used for continuous variables and chi-square tests for categorical variables. In order to evaluate the effects of the intervention over time, a repeated-measures analysis of variance with a Group × Time model was applied, considering group membership (experimental versus control) and assessment time (pre-intervention versus post-intervention) as factors. This approach was adopted to verify both the main effects of time and possible Group × Time interactions, considered indicative of differential changes between the experimental and control groups over the study period. Given the class-based structure of the study, exploratory intraclass correlation coefficients (ICCs) were estimated for the principal outcome variables in order to evaluate the potential influence of class membership. ICC values were generally low, indicating that only a limited proportion of the total variance was attributable to differences between classes. Therefore, the primary analyses were conducted at the individual student level, while the clustered nature of the data was considered when interpreting the findings.

Given the multidimensional nature of the intervention, the study included cognitive, physiological, nutritional, and academic outcome measures. Particular emphasis was placed on executive function and readiness for learning indicators, assessed through the Stroop Test, Digit Span performance, and the school engagement scale, which were considered the outcomes most directly aligned with the primary study objectives. Bonferroni correction was applied to post hoc comparisons in order to reduce the risk of Type I error associated with multiple testing. The remaining outcomes were interpreted as complementary indicators contributing to the overall evaluation of the intervention.

When necessary, post hoc analyses with Bonferroni correction were conducted for comparisons between experimental conditions. Furthermore, effect sizes were calculated using Cohen’s d and partial eta squared in order to provide a measure of the magnitude of the observed group differences beyond statistical significance alone. Cohen’s d values were calculated as standardized measures of within-group pre–post change and were reported as complementary indicators of the magnitude of change observed in the experimental group. The primary evaluation of intervention effectiveness was based on the Group × Time interaction obtained from the repeated-measures ANOVA, with partial eta squared (η^2^) and between-group differences in change scores with 95% confidence intervals used to quantify intervention effects. Cohen’s d values of 0.20, 0.50, and 0.80 were interpreted respectively as small, moderate, and large effects, whereas partial eta squared values of 0.01, 0.06, and 0.14 were considered indicative of small, moderate, and large effects.

Relationships between cognitive, physiological, and academic variables were further explored through Pearson correlation analysis. In particular, associations between changes in executive functions, cardiorespiratory fitness, school engagement, and academic achievement were analyzed. Internal consistency of the self-report scales used in the study was verified through Cronbach’s alpha coefficient. Prior to the main analyses, data were screened for completeness and potential outliers through visual inspection of boxplots and standardized residuals. No extreme outliers requiring exclusion were identified. All participants completed both baseline and post-intervention assessments, resulting in a complete dataset with no missing values for the primary study variables. Consequently, all analyses were conducted on the full sample of participants who completed the study (*n* = 110).

## 3. Results

### 3.1. Preliminary Descriptive Analyses

**Figure 1:**
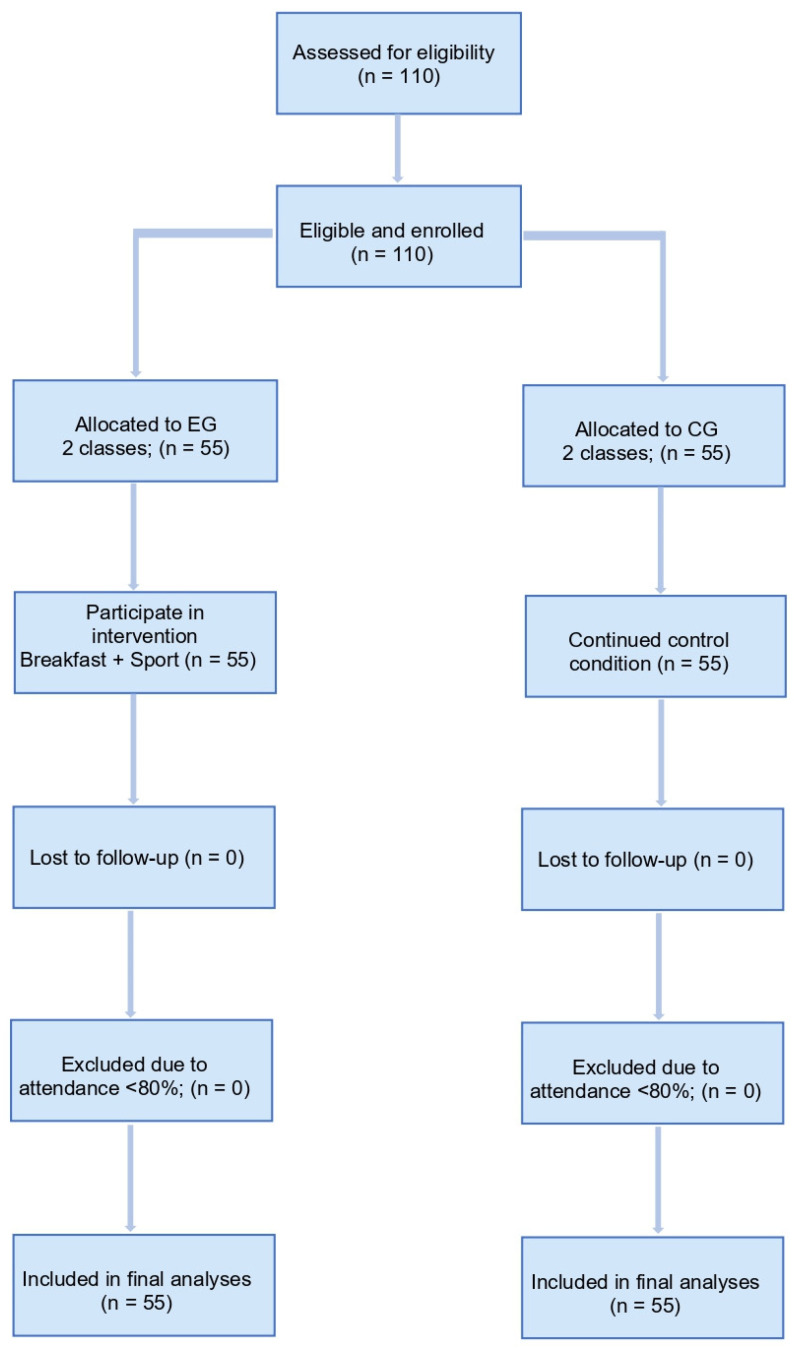
Study participants’ flow diagram.

All 110 enrolled participants completed both baseline and post-intervention assessments. No participants were excluded during the study period due to absence rates exceeding the predefined threshold, and no losses to follow-up occurred. Consequently, all participants allocated to the experimental group (*n* = 55) and control group (*n* = 55) were included in the final analysis (Figure 1).

Preliminary descriptive analyses (Table 3) were conducted in order to examine sample characteristics and verify data distribution. The final sample included 110 adolescents aged between 14 and 16 years, equally distributed between the experimental group (*n* = 55) and the control group (*n* = 55). The overall mean age of participants was 15.1 ± 0.7 years, with no significant differences between the experimental group (15.0 ± 0.6 years) and the control group (15.2 ± 0.7 years). Regarding sex distribution, the experimental group included 28 males (50.9%) and 27 females (49.1%), whereas the control group included 29 males (52.7%) and 26 females (47.3%).

Baseline anthropometric and physiological characteristics appeared generally homogeneous between groups. Mean body mass index values were 22.1 ± 2.8 kg/m^2^ in the experimental group and 22.4 ± 3.0 kg/m^2^ in the control group. Body weight status was additionally interpreted according to age- and sex-adjusted BMI percentiles for adolescents, based on World Health Organization growth reference standards. Resting heart rate values were 76.3 ± 7.4 bpm and 77.1 ± 6.9 bpm for the experimental and control groups, respectively. Mean performance in the 20 m shuttle run test corresponded to 5.8 ± 1.2 completed levels in the experimental group and 5.7 ± 1.1 levels in the control group.

Regarding cognitive variables, mean Stroop Test scores at baseline were 68.4 ± 10.2 in the experimental group and 69.1 ± 10.5 in the control group. Digit Span Forward scores showed mean values of 6.4 ± 1.0 for the experimental group and 6.3 ± 1.1 for the control group, whereas Digit Span Backward scores were 4.7 ± 0.9 and 4.6 ± 1.0, respectively.

Descriptive analyses of nutritional and academic variables indicated comparable baseline conditions between groups. Mean Breakfast Habits Score values were 2.8 ± 0.6 in the experimental group and 2.7 ± 0.5 in the control group. Mean school engagement scores at baseline were 3.1 ± 0.6 in the experimental group and 3.0 ± 0.5 in the control group. Average academic grades were 6.8 ± 0.7 and 6.7 ± 0.8, respectively.

Normality of data distribution was verified using the Shapiro–Wilk test. Most variables showed values consistent with normal distribution assumptions, with *p*-values above 0.05. Homogeneity of variances between groups was confirmed through Levene’s test, which did not reveal significant violations for the main study variables (*p* > 0.05). Consequently, parametric statistical procedures were considered appropriate for subsequent analyses.

### 3.2. Baseline Comparisons Between Experimental and Control Groups

Independent-samples *t*-tests and chi-square analyses (Table 4) were conducted to examine baseline differences between the experimental and control groups regarding anthropometric, cognitive, physiological, nutritional, and academic variables. The analysis revealed no statistically significant differences between groups at baseline, confirming the initial comparability of participants prior to the intervention.

With regard to anthropometric and physiological variables, no significant between-group differences emerged for body mass index, t(108) = −0.51, *p* = 0.612, resting heart rate, t(108) = −0.60, *p* = 0.548, or cardiorespiratory fitness assessed through the 20 m shuttle run test, t(108) = 0.36, *p* = 0.721. Similarly, cognitive baseline measures did not significantly differ between groups. Stroop Test scores were comparable between the experimental and control groups, t(108) = −0.38, *p* = 0.703, as were Digit Span Forward scores, t(108) = 0.42, *p* = 0.677, and Digit Span Backward scores, t(108) = 0.34, *p* = 0.734.

Regarding nutritional and academic variables, independent-samples *t*-tests showed no significant baseline differences in Breakfast Habits Score values, t(108) = 0.79, *p* = 0.433, school engagement scores, t(108) = 0.86, *p* = 0.392, or average academic grades, t(108) = 0.65, *p* = 0.514.

### 3.3. Intraclass Correlation Analysis

Exploratory intraclass correlation coefficients (ICCs) were calculated for the principal outcome variables to evaluate the extent to which class membership contributed to the observed variability. ICC values ranged from 0.02 to 0.08, indicating low to modest between-class variability. Specifically, ICCs were 0.03 for Stroop Test performance, 0.02 for Digit Span Backward, 0.02 for the 20 m shuttle run test, 0.04 for breakfast habits, 0.06 for school engagement, and 0.08 for academic achievement. These findings suggest that most of the observed variance was attributable to individual-level differences rather than to class-level clustering.

### 3.4. Changes over Time in the Experimental and Control Groups

A repeated-measures analysis of variance with a Group × Time model (Table 5) was conducted in order to evaluate the effects of the intervention over time, considering group membership (experimental versus control) and assessment time (pre-intervention versus post-intervention) as factors. The analysis revealed significant Group × Time interactions for several cognitive, physiological, nutritional, and academic variables, indicating differential changes between the experimental and control groups over the study period. For the principal outcomes, between-group differences in change scores and their corresponding 95% confidence intervals are reported in Table 4 to facilitate interpretation of the magnitude and precision of the observed intervention effects.

Regarding cognitive outcomes, a significant Group × Time interaction emerged for Stroop Test scores, F(1,108) = 11.94, *p* = 0.001, partial η^2^ = 0.100. In particular, the experimental group demonstrated greater improvements in Stroop Test performance over time, from 68.4 ± 10.2 at baseline to 59.8 ± 8.9 at post-intervention, whereas the control group showed only minimal changes from 69.1 ± 10.5 to 67.5 ± 9.8. No significant interaction effect was observed for Digit Span Forward scores, F(1,108) = 2.11, *p* = 0.149, partial η^2^ = 0.019, although the experimental group demonstrated a slight increase from 6.4 ± 1.0 at baseline to 6.8 ± 1.1 at post-intervention. However, a significant Group × Time interaction emerged for Digit Span Backward performance, F(1,108) = 7.42, *p* = 0.008, partial η^2^ = 0.064. Digit Span Backward scores increased in the experimental group from 4.7 ± 0.9 at baseline to 5.5 ± 1.0 post-intervention, whereas the control group showed only marginal changes from 4.6 ± 1.0 to 4.8 ± 1.0.

Significant Group × Time interactions were also observed for physiological variables. Cardiorespiratory fitness assessed through the 20 m shuttle run test showed greater increases in the experimental group than in the control group, F(1,108) = 10.91, *p* = 0.001, partial η^2^ = 0.092. Mean completed levels increased from 5.8 ± 1.2 to 7.0 ± 1.3 in the experimental group, while the control group showed only slight variation from 5.7 ± 1.1 to 5.9 ± 1.2. Resting heart rate also showed a significant interaction effect, F(1,108) = 5.87, *p* = 0.017, partial η^2^ = 0.052, with the experimental group demonstrating a reduction from 76.3 ± 7.4 bpm to 72.1 ± 6.8 bpm, whereas the control group remained substantially unchanged. No significant Group × Time interaction emerged for body mass index, F(1,108) = 1.36, *p* = 0.246, partial η^2^ = 0.012, indicating that no substantial anthropometric differences emerged between groups over the study period.

Regarding nutritional outcomes, the experimental group demonstrated significant improvements in Breakfast Habits Score values compared with the control group, F(1,108) = 13.28, *p* < 0.001, partial η^2^ = 0.110. Mean Breakfast Habits Score values increased from 2.8 ± 0.6 to 4.1 ± 0.5 in the experimental group, whereas the control group showed only limited changes from 2.7 ± 0.5 to 2.9 ± 0.5.

Academic and school engagement outcomes also showed significant improvements over time in the experimental group. A significant Group × Time interaction was found for school engagement scores, F(1,108) = 11.36, *p* = 0.001, partial η^2^ = 0.095. Mean school engagement values increased from 3.1 ± 0.6 to 3.8 ± 0.5 in the experimental group, whereas only minimal changes were observed in the control group. Academic grades showed a smaller but still significant interaction effect, F(1,108) = 4.94, *p* = 0.028, partial η^2^ = 0.044. In particular, the experimental group improved from an average grade of 6.8 ± 0.7 to 7.3 ± 0.6, while the control group remained relatively stable across the study period.

### 3.5. Post Hoc Analyses and Effect Sizes

Post hoc analyses (Table 6) with Bonferroni correction were conducted for variables showing significant Group × Time interactions in order to identify the specific differences between pre- and post-intervention conditions. The analysis confirmed that the experimental group demonstrated greater favorable changes across several cognitive, physiological, nutritional, and academic variables over the study period compared with the control group.

In particular, Bonferroni-adjusted comparisons revealed a significant improvement in Stroop Test scores in the experimental group between baseline and post-intervention assessments (*p* = 0.001). The magnitude of this change was moderate-to-large, with a Cohen’s d value of 0.81. Changes in Digit Span Forward scores did not reach statistical significance after correction for multiple comparisons (*p* = 0.149), showing only a small effect size (Cohen’s d = 0.21). Conversely, Digit Span Backward performance showed a significant post-intervention improvement in the experimental group (*p* = 0.008), with a moderate effect size (Cohen’s d = 0.63).

Regarding physiological variables, post hoc analyses confirmed significant improvements in cardiorespiratory fitness among participants in the experimental group, with the 20 m shuttle run test showing a moderate effect size (Cohen’s d = 0.71). Resting heart rate demonstrated a smaller but statistically significant reduction following the intervention (*p* = 0.017), corresponding to a small-to-moderate effect size (Cohen’s d = 0.48). No significant post-intervention changes were observed for body mass index (*p* = 0.246), confirming the absence of substantial anthropometric modifications during the study period.

School engagement scores significantly increased in the experimental group after the intervention (*p* = 0.001), with a moderate-to-large effect size (Cohen’s d = 0.76). Academic grades also showed a statistically significant increase following the intervention (*p* = 0.028), although the magnitude of change was smaller (Cohen’s d = 0.39), indicating a gradual change in academic performance over the study period. Nutritional outcomes showed meaningful changes in breakfast-related behaviors, with Breakfast Habits Score values significantly improving after the intervention (*p* < 0.001), corresponding to a large effect size (Cohen’s d = 0.88).

### 3.6. Correlation Analyses and Internal Consistency

Pearson correlation analyses were conducted in order to further explore the relationships among changes observed in cognitive, physiological, nutritional, and academic variables during the intervention period. Correlations were calculated using individual change scores (post-intervention minus pre-intervention values) within the experimental group. Associations between changes in executive functioning, cardiorespiratory fitness, breakfast habits, school engagement, and academic achievement were examined within the experimental group.

The analyses revealed significant moderate correlations between improvements in Stroop Test performance and school engagement scores (r = −0.46, *p* = 0.001), indicating that students showing greater improvements in attentional and inhibitory control abilities also tended to report higher levels of school participation and motivation. Improvements in Stroop Test scores were also moderately associated with improvements in average academic grades (r = −0.41, *p* = 0.003), indicating an association between changes in executive functioning and academic performance.

Digit Span Backward improvements demonstrated positive correlations with both school engagement (r = 0.38, *p* = 0.006) and academic achievement (r = 0.35, *p* = 0.011), indicating that greater improvements in working memory tended to co-occur with higher school engagement and academic achievement.

Cardiorespiratory fitness improvements assessed through the 20 m shuttle run test were positively correlated with school engagement scores (r = 0.43, *p* = 0.002) and moderately associated with improvements in academic grades (r = 0.31, *p* = 0.021). In addition, reductions in resting heart rate were weakly but significantly associated with improved Stroop Test performance (r = −0.29, *p* = 0.034), indicating an association between physiological and cognitive changes observed during the intervention period.

Regarding nutritional variables, improvements in Breakfast Habits Score values showed positive associations with school engagement (r = 0.40, *p* = 0.004) and attentional performance assessed through the Stroop Test (r = −0.37, *p* = 0.008). These findings indicate that positive changes in breakfast-related behaviors tend to co-occur with favorable changes in school engagement and attentional performance. Students demonstrating healthier breakfast habits also tended to report more stable academic performance throughout the intervention period.

No significant correlations emerged between body mass index changes and the main cognitive or academic variables, suggesting that, within the present sample, anthropometric changes were not significantly associated with the cognitive, behavioral, or academic variables examined.

**Figure 2:**
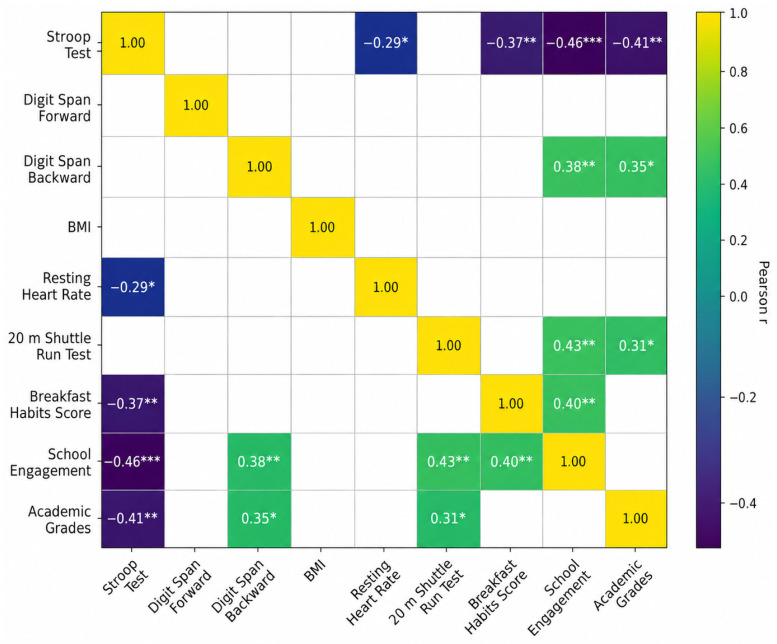
Pearson correlation heatmap showing the relationships among changes in cognitive, physiological, nutritional, and academic variables (post-intervention minus pre-intervention values) within the experimental group (*n* = 55). Blank cells indicate non-significant or non-reported associations. Note: Values represent Pearson correlation coefficients (r) calculated using individual change scores (post-intervention minus pre-intervention values) within the experimental group (*n* = 55). Negative correlations involving the Stroop Test scores and resting heart rate indicate that greater reductions in these variables were associated with more favorable cognitive or physiological outcomes. Lower Stroop Test scores indicate better performance because the composite score integrates completion time and number of errors across the test conditions. Blank cells indicate non-significant or non-reported associations. * *p* < 0.05, ** *p* < 0.01, and *** *p* ≤ 0.001.

Internal consistency analyses demonstrated acceptable reliability values for the self-report instruments used in the study. The Breakfast Habits Questionnaire showed a Cronbach’s alpha coefficient of 0.81, whereas the school engagement scale demonstrated a Cronbach’s alpha value of 0.86, indicating good internal consistency for both instruments (Figure 2).

### 3.7. Adherence to the Intervention

Adherence to the intervention was monitored throughout the study period using attendance registers and nutritional adherence diaries. Participants attended an average of 97% of the planned sessions, indicating a high level of engagement with the intervention protocol. Compliance with the breakfast component was also high, with 92% adherence to the prescribed nutritional plan across the intervention period. Most participants regularly consumed the meals provided according to the nutritional recommendations established by the supervising nutritionist. No major adverse events or difficulties related to breakfast consumption were reported during the intervention period.

No adverse events, injuries, or relevant complaints related to breakfast consumption or participation in the sport sessions were reported during the intervention period. In particular, no participants reported gastrointestinal discomfort, intolerance to the proposed meals, or difficulties in completing the scheduled activities.

## 4. Discussion

The present study was designed to investigate the effects of an integrated school-based intervention consisting of a structured breakfast and morning sport sessions on executive functions, readiness for learning, physiological well-being, and academic achievement among adolescent students. The results suggest that participation in a program combining nutritional support and cognitively oriented sport-based activities was associated with favorable changes in different dimensions involved in school learning processes, supporting the relevance of multidimensional approaches integrating health, sport, and education within the school context. One of the main findings concerns the greater improvement in cognitive performance observed in the experimental group relative to the control group, particularly regarding selective attention, inhibitory control, information processing speed, and working memory assessed through the Stroop Test and Digit Span Backward. Students in the experimental group demonstrated greater improvements in Stroop Test performance over time compared with baseline values and with the control group. This result may reflect the potential contribution of morning sport activities to neurophysiological activation and attentional efficiency during the early hours of the school day. The exercises proposed during the intervention required continuous motor adaptation, rapid responses to stimuli, selective attention, and control of environmental interference, components closely associated with executive functioning.

From a neurobiological perspective, moderate and cognitively demanding sport practice appears to promote increased cerebral blood flow and the release of neurotransmitters involved in attentional processes and cognitive regulation [41]. Furthermore, participation in sport activities before the beginning of lessons may have been associated with a more favorable activation state and greater cognitive readiness during subsequent school activities. These findings are consistent with international literature showing that sport-based interventions characterized by high coordinative and decisional demands may produce stronger effects on executive functions compared with exclusively repetitive or aerobic motor activities [42,43,44].

Digit Span Forward performance showed only limited and non-significant changes following the intervention. This finding suggests that participation in the integrated program may have been more strongly associated with attentional regulation and inhibitory control processes than with simple short-term verbal memory abilities. Considering the relatively brief duration of the intervention and the moderate intensity of the proposed cognitive demands, it is plausible that participation in the program was associated with changes in cognitive readiness and executive functioning rather than producing substantial modifications in passive memory span performance [45,46]. Conversely, Digit Span Backward performance showed significant improvement following the intervention, suggesting a stronger association with working memory processes requiring active manipulation and updating of information. This distinction may indicate that cognitively engaging sport-based activities exert stronger effects on executive components of memory rather than on passive verbal span abilities [47]. Nevertheless, the slight positive trend observed in the experimental group may indicate a potential contribution of cognitively engaging sport activities to short-term information processing during adolescence.

Several studies conducted during developmental age have shown that structured sport participation may positively influence cognitive functioning, particularly when activities involve coordinative demands, rapid decision making, and continuous adaptation to environmental stimuli [48,49]. However, evidence regarding short-term memory improvements appears less consistent compared with findings related to attention and inhibitory control. In contrast, working memory tasks involving executive control and active cognitive manipulation appear more sensitive to cognitively demanding sport interventions, particularly during adolescence [50]. Moreover, nutritional mechanisms may have contributed to the observed findings by supporting cerebral energy availability during morning hours and facilitating attentional and cognitive processes involved in school performance [51].

Regarding physiological aspects, the experimental group showed improvements in cardiorespiratory fitness and a slight reduction in resting heart rate at the end of the intervention. These results may be associated with regular participation in moderate sport-based activities carried out throughout the study period. Although the program was not specifically oriented toward athletic performance, the systematic repetition of intermittent aerobic activities, coordinative exercises, and dynamic games may have produced physiological adaptations sufficient to improve students’ general cardiovascular efficiency. No substantial changes were observed in body mass index values, suggesting that the duration of the intervention was not sufficient to produce relevant anthropometric modifications.

From a theoretical perspective, improved cardiorespiratory efficiency may also have indirectly contributed to the observed cognitive improvements [50]. Scientific literature has frequently highlighted positive relationships between aerobic fitness, cerebral perfusion, and executive functioning in adolescents. Some authors suggest that higher fitness levels may promote greater cerebral oxygenation and more favorable metabolic conditions for cognitive functioning, with positive effects on attention, self-regulation, information processing speed, and working memory performance [52]. The findings obtained in the present study appear to fit coherently within this interpretative framework.

Another relevant finding concerns the improvement observed in breakfast habits among students belonging to the experimental group. At the end of the intervention, students demonstrated healthier and more regular breakfast behaviors compared to baseline values and those of the control group. This result may reflect the combination of direct practical experience, school supervision, and the progressive acquisition of greater awareness regarding the importance of nutrition for well-being and school performance [53].

Integrating a structured breakfast into the school routine may have been associated not only with healthier eating behaviors, but also with more conscious attitudes toward nutrition [54]. The literature also emphasizes that breakfast regularity is frequently associated with better attention levels, lower cognitive fatigue, and greater emotional stability during the school day, aspects that may have indirectly contributed to the cognitive and academic improvements observed in the present study [55].

Regarding academic outcomes, students in the experimental group showed moderate improvement in academic achievement and higher levels of school engagement compared with the control group. Although changes in school grades were relatively modest (Cohen’s d = 0.39), the observed trend may indicate that participation in the program was associated with more favorable conditions for learning and school participation during the intervention period. Improvements in motivation, attention during lessons, and perceived involvement in classroom activities represent some of the most interesting findings emerging from the study.

These results may be explained by considering that readiness for learning does not depend exclusively on individual cognitive competencies, but also on physiological, motivational, and emotional factors influencing readiness to learn [56]. Within this perspective, sport-based educational approaches may represent important tools for promoting inclusion, participation, and overall well-being in school contexts [57]. The combination of morning sport activities and adequate nutritional support may have been associated with better attentional regulation and a more favorable psychological predisposition toward school activities. Furthermore, the collaborative and dynamic nature of the sport-based activities may have been associated with higher levels of perceived belonging, self-efficacy, and social involvement, positively influencing everyday school participation [58].

Participation in structured sport-based activities appears to promote self-regulation processes, stress management, cooperation, intrinsic motivation, and working memory engagement, all factors closely related to the quality of school experience and academic achievement [59,60,61]. Overall, the findings of the present study suggest that participation in integrated school-based programs combining nutrition and sport-based activities may be associated with favorable changes in cognitive functioning and academic outcomes during adolescence. Addressing physiological, cognitive, and behavioral dimensions simultaneously appears particularly relevant during a developmental phase characterized by significant changes in lifestyle habits, daily rhythms, and school demands.

However, the study also presents some limitations that should be considered when interpreting the results. First, the sample was recruited from a single school located in Southern Italy, limiting the generalizability of the findings to other geographical, cultural, and educational contexts. Furthermore, although the quasi-experimental design ensured high ecological validity, the use of pre-existing school classes rather than individual randomization limited the possibility of fully controlling all factors that may have influenced educational outcomes. Students were nested within intact classes and, despite the relatively low intraclass correlation coefficients observed for the principal outcome variables, some class-level influences cannot be entirely excluded. Although exploratory ICC analyses suggested a limited clustering effect, the class-based allocation procedure may have reduced the effective sample size compared with an individually randomized design and should therefore be considered when interpreting the findings. Therefore, the findings should be interpreted as associations between participation in the program and changes over time rather than as definitive evidence of causal effects. An additional limitation concerns the multicomponent nature of the intervention. Because the program simultaneously combined a structured breakfast and cognitively oriented sport activities, it was not possible to determine the relative contribution of each component to the observed outcomes. Consequently, the findings should be interpreted as reflecting participation in the overall integrated program rather than the isolated effects of nutritional support or sport activities. Furthermore, it cannot be excluded that factors such as increased adult supervision, greater routine structure, or other contextual characteristics of the intervention may have contributed to the observed changes. Similarly, because the control group continued its usual school activities without receiving an attention-matched condition, expectancy and attention-related effects may have partially influenced some outcomes, particularly school engagement and self-reported breakfast habits. Some measures, particularly those related to breakfast habits and school engagement, were based on self-report instruments and may therefore have been influenced by subjective bias or social desirability. In addition, although alternative stimulus sequences were adopted for repeated cognitive assessments, potential practice effects associated with repeated testing cannot be completely excluded. Furthermore, exercise intensity was monitored through perceived exertion ratings rather than objective physiological measures, and nutritional adherence was evaluated through dietary records rather than direct quantification of nutrient intake. Another limitation concerns the absence of information regarding potentially relevant contextual and behavioral variables that may have influenced the observed outcomes. In particular, sleep duration and sleep quality were not assessed, despite their recognized role in cognitive functioning, readiness for learning, and attentional performance during adolescence. Similarly, socioeconomic status, parental educational level, screen time, participation in extracurricular sport activities, and habitual lifestyle behaviors outside the intervention were not systematically monitored. Although baseline assessments indicated comparable conditions between groups for the principal study variables, the potential influence of these unmeasured factors cannot be entirely excluded and should be considered when interpreting the findings. Moreover, academic grades are influenced by multiple educational, instructional, and contextual factors, and should therefore be interpreted as complementary educational indicators rather than standardized measures of cognitive performance. Finally, the relatively limited duration of the intervention may not have been sufficient to produce larger changes in academic achievement or anthropometric indicators. Future studies involving a larger number of schools and classes, longer follow-up periods, multilevel analytical approaches, and factorial or multi-arm designs are needed to strengthen the generalizability and robustness of the findings and to clarify the specific contribution of the different intervention components.

Despite these limitations, the present study also presents important strengths. In particular, the intervention was developed within a real school setting, using an integrated approach combining nutrition, sport, and cognitive processes within everyday educational routines. The multidimensional nature of the adopted measures allowed the simultaneous analysis of cognitive, physiological, nutritional, and academic aspects, offering a broader perspective compared with many previous studies focusing exclusively on single variables. Furthermore, the choice of cognitively oriented sport-based activities represents an innovative element, as it overcomes an exclusively quantitative view of physical activity by emphasizing instead the cognitive and coordinative quality of the sport experience.

## 5. Conclusions

In recent years, growing attention toward the relationship between health, learning, and cognitive functioning has highlighted the need to develop multidimensional school-based interventions capable of integrating nutrition, sport, and educational processes. Adolescence represents a particularly sensitive developmental phase for the promotion of healthy habits and for the enhancement of cognitive and self-regulatory competencies closely related to academic success. Within this context, the present study examined the associations between participation in an integrated program based on a structured breakfast and cognitively oriented morning sport sessions and a range of cognitive, physiological, behavioral, and academic outcomes within the school environment.

The findings suggest that participation in a program combining nutritional support and sport-based activities was associated with favorable changes in executive functioning, cardiorespiratory fitness, school engagement, and breakfast habits among adolescents. In particular, the observed improvements in attentional processes, inhibitory control, and working memory performance suggest the potential educational value of interventions designed to support neurophysiological activation, cognitive functioning, and readiness for learning. Conversely, changes in simple short-term verbal memory performance and anthropometric indicators appeared limited, suggesting that the observed changes were more evident in cognitive, behavioral, and physiological dimensions than in broader anthropometric indicators. Although the effects on academic achievement were moderate, the observed trend is consistent with the hypothesis that more favorable physiological and cognitive conditions may be associated with improvements in the quality of school experience and educational performance.

From an applied perspective, the study findings highlight the importance of reconsidering the role of schools as active environments for the promotion of students’ health and overall well-being. The integration of nutritional programs and morning sport-based activities within the school routine appears feasible within the specific educational context examined in the present study and may be associated with improvements in physical well-being, school participation, attentional regulation, working memory processes, and learning-related outcomes. However, aspects related to feasibility, acceptability, organizational requirements, resource allocation, and scalability were not directly evaluated and should therefore be explored in future research before broader implementation can be recommended. Furthermore, further studies involving larger samples, longitudinal follow-ups, different educational contexts and randomized controlled designs will be necessary to verify the stability and generalizability of the observed findings. Taken together, the findings of the present study contribute to expanding the available evidence regarding integrated school-based programs, emphasizing the potential value of interdisciplinary educational approaches aimed at supporting healthy development, cognitive functioning, and academic engagement during adolescence.

## Figures and Tables

**Table 1:** Example of weekly structure of the integrated intervention program.

Day	Intervention Sequence	Nutritional Plan	Initial Phase Activities	Central Phase Activities	Final Phase Activities	Main Objectives
Day 1	Structured breakfast → transition → sport session → regular lessons	Milk or yogurt, whole-grain bread with honey, fresh fruit, and water	Joint mobility, light running, and coordinative drills	Coordinative pathways with changes in direction, ladder drills, speed exercises based on visual and auditory signals, and motor tasks associated with recognition of colors and numbers	Dynamic stretching, controlled breathing and active cool-down	Selective attention, coordination, and processing speed
Day 2	Structured breakfast → transition → sport session → regular lessons	Greek yogurt or milk, whole-grain cereals, dried fruit, a banana or apple, and water	Dynamic balance exercises and general motor activation	Small-sided games, collaborative pair activities, simplified sport games with variable rules, and motor decision-making exercises	Dynamic stretching, attentional relaxation, and guided breathing	Decision-making speed, cognitive adaptation, and attentional control
Day 3	Structured breakfast → transition → sport session → regular lessons	Toasted whole-grain bread, low-fat ricotta cheese, fresh fruit, and water	Dynamic drills, general mobility, and coordinative exercises	Moderate-intensity intermittent circuits, station activities, and aerobic exercises integrated with working memory tasks and rapid responses to verbal and visual stimuli	Active cool-down, stretching, and breathing regulation	Neurophysiological activation, executive functioning, and attentional regulation

**Table 2:** Nutritional characteristics of the structured breakfast program.

Day	Main Food Components	Approx. Energy (kcal)	Carbohydrates (%)	Protein (%)	Fat (%)	Fiber (g)	Hydration (mL)
Day 1	Milk or plain yogurt, whole-grain bread, honey or sugar-free jam, and fresh fruit	380–420	55–60	15–18	25–30	6–8	250–300
Day 2	Greek yogurt or milk, whole-grain cereals, dried fruit, and a banana or apple	400–450	50–55	18–20	25–30	7–9	250–300
Day 3	Toasted whole-grain bread, low-fat ricotta cheese, and fresh fruit	380–430	50–55	18–20	25–30	6–8	250–300

**Table 3:** Baseline descriptive characteristics of experimental and control groups.

Variable	EG (*n* = 55) Mean ± SD/*n* (%)	CG (*n* = 55) Mean ± SD/*n* (%)	*p*-Value
Age (years)	15.0 ± 0.6	15.2 ± 0.7	0.184
Male participants	28 (50.9%)	29 (52.7%)	0.851
Female participants	27 (49.1%)	26 (47.3%)	0.851
Body mass index (kg/m^2^)	22.1 ± 2.8	22.4 ± 3.0	0.612
Resting heart rate (bpm)	76.3 ± 7.4	77.1 ± 6.9	0.548
20 m shuttle run test (levels)	5.8 ± 1.2	5.7 ± 1.1	0.721
Stroop Test score	68.4 ± 10.2	69.1 ± 10.5	0.703
Digit Span Forward	6.4 ± 1.0	6.3 ± 1.1	0.677
Digit Span Backward	4.7 ± 0.9	4.6 ± 1.0	0.734
Breakfast Habits Score	2.8 ± 0.6	2.7 ± 0.5	0.433
School engagement score	3.1 ± 0.6	3.0 ± 0.5	0.392
Average academic grades	6.8 ± 0.7	6.7 ± 0.8	0.514

Note. Lower scores indicate better performance. Stroop Test scores were calculated using a composite index based on completion time and number of errors across the test conditions.

**Table 4:** Baseline comparisons between experimental and control groups.

Variable	EG Mean ± SD/*n* (%)	C Mean ± SD/*n* (%)	Test Statistic	*p*-Value
Body mass index (kg/m^2^)	22.1 ± 2.8	22.4 ± 3.0	t = −0.51	0.612
Resting heart rate (bpm)	76.3 ± 7.4	77.1 ± 6.9	t = −0.60	0.548
20 m shuttle run test (levels)	5.8 ± 1.2	5.7 ± 1.1	t = 0.36	0.721
Stroop Test score	68.4 ± 10.2	69.1 ± 10.5	t = −0.38	0.703
Digit Span Forward	6.4 ± 1.0	6.3 ± 1.1	t = 0.42	0.677
Digit Span Backward	4.7 ± 0.9	4.6 ± 1.0	t = 0.34	0.734
Breakfast Habits Score	2.8 ± 0.6	2.7 ± 0.5	t = 0.79	0.433
School engagement score	3.1 ± 0.6	3.0 ± 0.5	t = 0.86	0.392
Average academic grades	6.8 ± 0.7	6.7 ± 0.8	t = 0.65	0.514

Note. Lower scores indicate better performance. Stroop Test scores were calculated using a composite index based on completion time and number of errors across the test conditions.

**Table 5:** Repeated-measures ANOVA results for Group × Time interactions in cognitive, physiological, and academic variables.

Variable	EG Pre Mean ± SD	EG Post Mean ± SD	EG Change (Δ)	CG Pre Mean ± SD	CG Post Mean ± SD	CG Change (Δ)	Between-Group Difference in Change (95% CI)	F (Group × Time)	*p* Value	Partial η^2^
Stroop Test score	68.4 ± 10.2	59.8 ± 8.9	−8.6	69.1 ± 10.5	67.5 ± 9.8	−1.6	−7.0 (−11.4, −2.6)	11.94	0.001	0.100
Digit Span Forward	6.4 ± 1.0	6.8 ± 1.1	+0.4	6.3 ± 1.1	6.4 ± 1.0	+0.1	0.3 (−0.2, 0.8)	2.11	0.149	0.019
Digit Span Backward	4.7 ± 0.9	5.5 ± 1.0	+0.8	4.6 ± 1.0	4.8 ± 1.0	+0.2	0.6 (0.2, 1.0)	7.42	0.008	0.064
Body mass index (kg/m^2^)	22.1 ± 2.8	22.0 ± 2.7	−0.1	22.4 ± 3.0	22.3 ± 2.9	−0.1	0.0 (−0.4, 0.4)	1.36	0.246	0.012
Resting heart rate (bpm)	76.3 ± 7.4	72.1 ± 6.8	−4.2	77.1 ± 6.9	76.5 ± 6.7	−0.6	−3.6 (−6.3, −0.9)	5.87	0.017	0.052
20 m shuttle run test (levels)	5.8 ± 1.2	7.0 ± 1.3	+1.2	5.7 ± 1.1	5.9 ± 1.2	+0.2	1.0 (0.4, 1.6)	10.91	0.001	0.092
Breakfast Habits Score	2.8 ± 0.6	4.1 ± 0.5	+1.3	2.7 ± 0.5	2.9 ± 0.5	+0.2	1.1 (0.7, 1.5)	13.28	<0.001	0.110
School engagement score	3.1 ± 0.6	3.8 ± 0.5	+0.7	3.0 ± 0.5	3.1 ± 0.5	+0.1	0.6 (0.3, 0.9)	11.36	0.001	0.095
Average academic grades	6.8 ± 0.7	7.3 ± 0.6	+0.5	6.7 ± 0.8	6.8 ± 0.7	+0.1	0.4 (0.1, 0.7)	4.94	0.028	0.044

Note. Change scores (Δ) were calculated as post-intervention minus pre-intervention values. Between-group differences in change were calculated as the difference between experimental group and control group change scores and are reported with corresponding 95% confidence intervals. Lower scores indicate better performance for the Stroop Test score and resting heart rate. Stroop Test scores were calculated using a composite index based on completion time and number of errors across the test conditions. Academic grades were expressed according to the Italian grading system ranging from 1 to 10, with 6 representing the minimum passing grade.

**Table 6:** Post hoc comparisons and effect size analyses for the main study variables in the experimental group.

Variable	Post Hoc Comparison	*p*-Value	Cohen’s d	Effect Size Interpretation
Stroop Test score	Pre- vs. Post-Experimental Group	0.001	0.81	Moderate-to-large
Digit Span Forward	Pre- vs. Post-Experimental Group	0.149	0.21	Small
Digit Span Backward	Pre- vs. Post-Experimental Group	0.008	0.63	Moderate
Body mass index (kg/m^2^)	Pre- vs. Post-Experimental Group	0.246	0.12	Small
Resting heart rate	Pre- vs. Post-Experimental Group	0.017	0.48	Small-to-moderate
20 m shuttle run test	Pre- vs. Post-Experimental Group	0.001	0.71	Moderate
Breakfast Habits Score	Pre- vs. Post-Experimental Group	<0.001	0.88	Large
School engagement score	Pre- vs. Post-Experimental Group	0.001	0.76	Moderate-to-large
Average academic grades	Pre- vs. Post-Experimental Group	0.028	0.39	Small-to-moderate

Note. Cohen’s d values were calculated as within-group effect sizes for pre–post changes in the experimental group using the pooled standard deviation of the pre- and post-intervention scores. Values of 0.20, 0.50, and 0.80 were interpreted as small, moderate, and large effects, respectively. Effect sizes are presented to facilitate interpretation of the magnitude of the observed changes beyond statistical significance.

## Data Availability

The datasets generated and analyzed during the current study are available from the corresponding author upon reasonable request. The data are not publicly available due to privacy restrictions.
